# Reverse sphincterotomy assisted accessory pancreatic duct cannulation

**DOI:** 10.1055/a-2608-0621

**Published:** 2025-06-18

**Authors:** Fan Fan, Jin-Hui Yi, Qian Tong, Liang-Hao Hu

**Affiliations:** 1 Department of Gastroenterology, Peking University People’s Hospital, Beijing, China; 2 Department of Gastroenterology, Shanghai Changhai Hospital, Naval Medical University, Shanghai, China; 3 12520 Digestive Endoscopy Center, Changhai Hospital, Naval Medical University, Shanghai, China

A 61-year-old woman admitted to our hospital with pancreatic duct stones. She underwent extracorporeal shockwave lithotripsy followed by endoscopic retrograde cholangiopancreatography (ERCP). Pancreatography demonstrated significant tortuosity of the main pancreatic duct (MPD) ( Fig. 1 **a** ) and acute angle between the ventral and proximal pancreatic duct which created technical difficulty in advancing the guidewire into the proximal pancreatic duct ( Fig. 1 **b** ). In this context, we attempted cannulation via minor papilla. However, the inconspicuous orifice of the minor papilla rendered cannulation challenging.

**Fig. 1:**
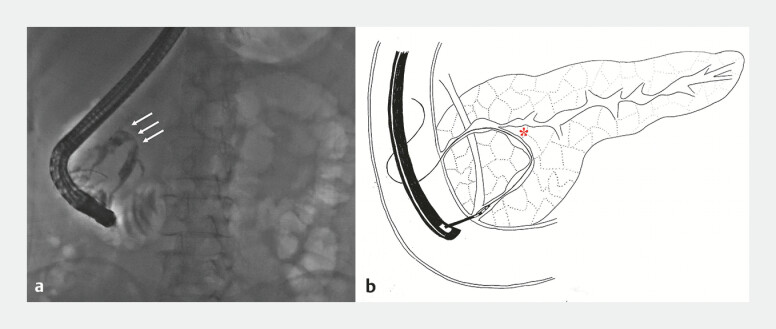
**a** Pancreatography demonstrated significant tortuosity of the main pancreatic duct (marked by the white arrows). **b** A schematic diagram demonstrating the acute angle between the ventral and proximal pancreatic duct (marked by the *) and showing of passage of the guidewire through the main pancreatic duct into the accessory pancreatic duct and subsequent retrograde passage through the minor papilla.

Ultimately, we advanced the guidewire retrogradely through the MPD into the accessory pancreatic duct (APD) and finally into the duodenal lumen via the minor papilla ( Fig. 2 **a** ). A sphincterotome was then passed retrogradely through a minor papilla along the guidewire. Reverse sphincterotomy was performed with the orientation toward 6–8 o’clock and enabled cannulation without any intraoperative complications ( Fig. 2 **b, c** ). Successful cannulation of APD was achieved through minor papilla ( Fig. 3 **a** ). Following dilation of APD with a balloon catheter ( Fig. 3 **b** ), the stones were extracted with a balloon extractor and a retrieval basket ( Fig. 3 **c** ). Two plastic stents (10 Fr, 5 cm and 7 Fr, 10 cm) were subsequently placed into APD ( Fig. 3 **d** ). The patient was observed for 2 days without any complications. And, no abdominal pain was revealed at 1-month follow-up.

**Fig. 2:**
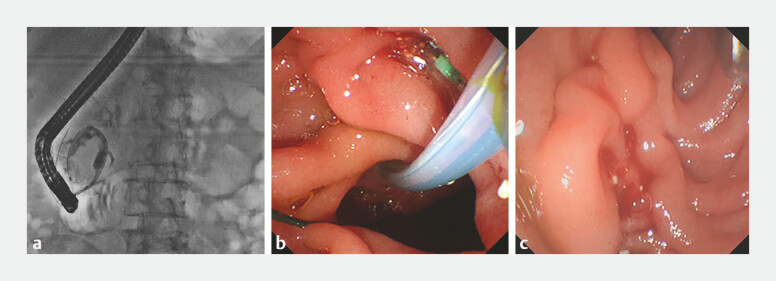
**a** The guidewire passed retrogradely through the main pancreatic duct into the accessory pancreatic duct and coiled in the duodenal lumen. **b** A sphincterotome passed reversely through the minor papilla along the guidewire and performed reverse sphincterotomy. **c** The orifice of minor papilla after reverse sphincterotomy.

**Fig. 3:**
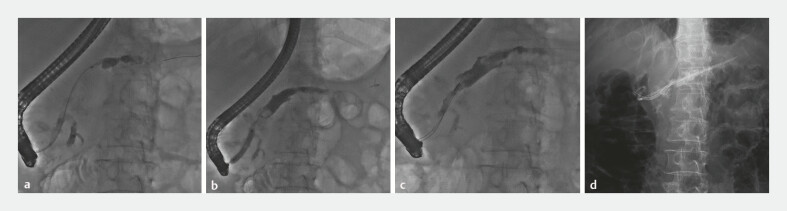
**a** Pancreatography showed successful cannulation through the accessory pancreatic duct. **b** A balloon catheter was used to dilate the accessory pancreatic duct. **c** A retrieval basket was used to clear the pancreatic duct stones. **d** Two plastic stents (10 Fr, 5 cm and 7 Fr, 10 cm) were placed in the accessory pancreatic duct.

ERCP is the first-line treatment for chronic pancreatitis (CP) patients 1 2 3 . However, cannulation of MPD can sometimes be difficult in cases such as pancreas divisum, MPD distortion, or obstruction due to pancreatic duct stones. In such scenarios, cannulation of APD serves as an alternative approach 4 . According to the experience of our center, approximately 20% of CP patients achieved treatment through minor papilla. Nevertheless, APD cannulation can be challenging if the orifice of minor papilla is inconspicuous. In this case, we employed a reverse sphincterotomy technique to facilitate the cannulation of minor papilla in a patient with CP, MPD distortion, and an inconspicuous accessory papillary orifice ( Video 1 ).

Reverse sphincterotomy assisted accessory pancreatic duct cannulation.Video 1

Endoscopy_UCTN_Code_TTT_1AR_2AC

## Notes

Endoscopy E-Videos https://eref.thieme.de/e-videos
*E-Videos* is an open access online section of the journal *Endoscopy* , reporting on interesting cases and new techniques in gastroenterological endoscopy. All papers include a high-quality video and are published with a Creative Commons CC-BY license. Endoscopy E-Videos qualify for HINARI discounts and waivers and eligibility is automatically checked during the submission process. We grant 100% waivers to articles whose corresponding authors are based in Group A countries and 50% waivers to those who are based in Group B countries as classified by Research4Life (see: https://www.research4life.org/access/eligibility/ ). This section has its own submission website at https://mc.manuscriptcentral.com/e-videos .
